# Is this scaling nonlinear?

**DOI:** 10.1098/rsos.150649

**Published:** 2016-07-13

**Authors:** J. C. Leitão, J. M. Miotto, M. Gerlach, E. G. Altmann

**Affiliations:** Max Planck Institute for the Physics of Complex Systems, Dresden, Germany

**Keywords:** scaling laws, statistical inference, allometry

## Abstract

One of the most celebrated findings in complex systems in the last decade is that different indexes *y* (e.g. patents) scale nonlinearly with the population *x* of the cities in which they appear, i.e. *y*∼*x*^*β*^,*β*≠1. More recently, the generality of this finding has been questioned in studies that used new databases and different definitions of city boundaries. In this paper, we investigate the existence of nonlinear scaling, using a probabilistic framework in which fluctuations are accounted for explicitly. In particular, we show that this allows not only to (i) estimate *β* and confidence intervals, but also to (ii) quantify the evidence in favour of *β*≠1 and (iii) test the hypothesis that the observations are compatible with the nonlinear scaling. We employ this framework to compare five different models to 15 different datasets and we find that the answers to points (i)–(iii) crucially depend on the fluctuations contained in the data, on how they are modelled, and on the fact that the city sizes are heavy-tailed distributed.

## 1. Introduction

The study of statistical and dynamical properties of cities from a complex-systems perspective is increasingly popular [1]. A celebrated result is the scaling between a city-specific observation *y* (e.g. the number of patents filed in the city) and the population *x* of the city as [2]

1.1 $$y=\alpha x^{\beta },$$

with a non-trivial (*β*≠1) exponent. Super-linear scaling (*β*>1) was observed when *y* quantifies creative or economic outputs and indicates that the concentration of people in large cities leads to an increase in the *per capita* (*y*/*x*) production. Sublinear scaling (*β*<1) was observed when *y* quantifies resource use and suggests that large cities are more efficient in the *per capita* (*y*/*x*) consumption. Since its proposal, nonlinear scaling has been reported in an impressive variety of different aspects of cities [3–9]. It has also inspired the proposal of different generative processes to explain its ubiquitous occurrence [10–14]. Scalings similar to the one in equation (1.1) appear in physical (e.g. phase transitions) and biological (e.g. allometric scaling) systems suggesting that cities share similarities with these and other complex systems (e.g. fractals).

More recent results cast doubts on the significance of the *β*≠1 observations [15–17]. Reference [15] agrees that economic outputs are faster than linear in *x*, but claims that the population *x* has a limited explanatory factor on the *per capita* rate *y*/*x* of cities and function (1.1) is not better than alternative ones (see [6,18] for opposing arguments). Reference [16] focuses on the case of CO_2_ emissions and shows that depending on whether city boundaries or metropolitan areas are used, the value of *β* changes from *β*>1 to *β*<1. This point was carefully analysed in [17] for different datasets *y*. Through a careful study of different possible choices of city boundaries, the authors report that the evidence for *β*≠1 virtually vanishes. These results ask for a more careful statistical analysis that rigorously quantifies the evidence for *β*≠1 in different datasets.

In this paper, we propose a statistical framework based on a probabilistic formulation of the scaling law (1.1) that allows us to perform hypothesis testing and model comparison. In particular, we quantify the evidence in favour of *β*≠1 comparing (through the Bayesian information criterion, BIC) models with *β*≠1 to models with *β*=1. We apply this approach to 15 datasets of cities from five regions and find that the conclusions regarding *β* vary dramatically not only depending on the datasets, but also on assumptions of the models that go beyond (1.1). We argue that the estimation of *β* is challenging and depends sensitively on the model because of the following two statistical properties of cities:

(i) the distribution of city population has heavy tails (Zipf’s law) [1,19] and
(ii) there are large and heterogeneous fluctuations of *y* as a function of *x* (heteroscedasticity).

**Figure 1.**
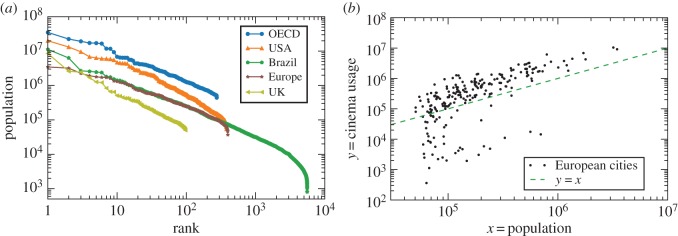
Example of the data and their main statistical properties. (*a*) The distribution of the population of the cities for the regions considered in this paper. The roughly straight line in this rank–population plot is in agreement with Zipf’s law and shows that, in most cases, the data vary over two orders of magnitude in the population (e.g. from 100 000 to 10 million inhabitants). (*b*) Example of the dataset analysed in our work, in which large fluctuations are clearly visible.

Points (i) and (ii) are shown, respectively, in figure 1*a*,*b*.

The paper is organized as follows. We start by describing the problem and the datasets we use (in §2) and discussing (in §3) the limitations of the usual statistical approach based on least-squares fitting in log-scale. We then propose a probabilistic formulation together with different statistical models (in §4) and describe (in §5) how they can be compared with each other and to data. Finally, we discuss our main findings (in §6) and summarize our conclusions (in §7).

## 2. Data

The general problem we are interested in is to test and estimate the parameters of equation (1.1) based on observations (*x*_*i*_,*y*_*i*_) for *i*=1,…,*N* cities, where *x*_*i*_ is the population and *y*_*i*_ is the amount of the quantity of interest in city *i* (as in figure 1*b*). The quantities *x*_*i*_,*y*_*i*_ are estimated within a measurement precision which, in principle, could also be included in the analysis. However, in most cases, this information is not available, and only single measurements of *x*_*i*_,*y*_*i*_ exist. The datasets we choose include a variety of different regions, aggregation methods to define city boundaries, and quantities *y*. They include the data from various countries and regions: 100 metropolitan areas of the United Kingdom (UK), aggregated as in [17]; 381 metropolitan areas of the United States of America (USA), as discussed in [12]; 459 urban areas of the USA; 472 large cities of the European Union (EU); 275 large cities from the members of the Organization for Economic Co-operation and Development (OECD); and 5565 municipalities (administrative units) from Brazil. For each database, we use indexes of economical activity (weekly income, GDP), innovation (patents filed), transportation (miles travelled, number of train stations), access to culture (number of theatres, number of cinema seats, number of cinema attendances in 1 year, etc.) and health condition (AIDS infections, death by external causes). Further details are presented in appendix A.

## 3. Limitations of the usual statistical analysis

The following three steps summarize the usual approach used to test a nonlinear scaling in equation (1.1) (see [2–4,8,11,12,16–18] for scalings in cities and [20] for scalings in biology):

1. The parameters of equation (1.1) are chosen based on least-squares fitting in log-transformed data ln⁡y,ln⁡x, i.e. *α*,*β* are such that $\sum_{i=1}^{N}{\left(\ln\alpha x_{i}^{\beta }-\ln y_{i}\right)}^{2}$ is minimized.
2. The quality of the fitting is quantified by the coefficient of determination $R^{2}\equiv 1-(\sum_{i}{\left(\ln y_{i}-\ln\alpha x_{i}^{\beta }\right)}^{2})/(\sum_{i}{\left(\ln y_{i}-\sum_{j}\ln y_{j}/N\right)}^{2})$. *R*^2^ close to 1 is taken as evidence of the agreement between the fit and the data.
3. The 95% confidence interval [*β*_min_,*β*_max_] around *β* is computed from the sum of the residuals squared and *β*∉[*β*_min_,*β*_max_] is taken as an evidence that *β*≠1.

This usual approach is appealing because of its simplicity and ease of numerical implementation. However, it contains the following assumptions and limitations that are usually ignored:

1. The parameters obtained through least-squares fitting are maximum-likelihood estimators if (i) the data points are independent and (ii) the fluctuations around the mean ln⁡y, ln⁡α+βln⁡x, are Gaussian distributed in ln⁡y with a variance independent of ln⁡x. The value of *β* obtained in the usual approach is meaningful if these assumptions hold.
2. *R*^2^ does not quantify the statistical significance of the model, it quantifies the correlation between data and model (the amount of the variation in the data explained by the model). In particular, *R*^2^ close to one is not an evidence that the data are a likely outcome of the model. Below, we obtain that datasets are typically not consistent with the model underlying the usual approach.
3. The confidence interval [*β*_min_,*β*_max_] is a range in which the true value of *β* is expected to be found only if the model holds [21]. Therefore, in the typical case in which the data are not compatible with the model, one cannot conclude that *β*≠1 based on the observation that 1∉[*β*_min_,*β*_max_]. Usually, in this case, both *β*=1 and *β*≠1 are incompatible with the data.
4. A further limitation of the usual approach is that it requires removing the datapoints with *y*_*i*_=0 (because it requires computing $\ln y_{i}$). This filtering is arbitrary, because *y*=0 is usually a valid observation (e.g. cities without any patents filed).

In the study of scaling laws in biology, the underlying hypothesis and alternatives to the usual least-squares fitting have been extensively discussed [22,23]. In city data, statistical analysis beyond the usual approach was performed in [3,5,7,8,10]. It typically amounts to an analysis of the residuals $\ln\alpha x_{i}^{\beta }-\ln y_{i}$, e.g. a (visual) comparison of the residuals of the fit to the Gaussian distribution predicted by the model underlying the linear fit in log–log scale. The controversies regarding a nonlinear scaling *β*≠1 motivate us to search for an alternative statistical framework to test the scaling (1.1) beyond the usual approach with residual analysis.

## 4. Probabilistic models

The statistical analysis we propose is based on the likelihood L of the data being generated by different models. Following [5], we assume that the index *y* (e.g. number of patents) of a city of size *x* is a random variable with probability density *P*(*y*|*x*). We interpret equation (1.1) as the scaling of the expectation of *y* with *x*

4.1 $$E(y|x)=\alpha x^{\beta },$$

where E(f(y)∣x)≡∫f(y)P(y∣x) dy is computed over the ensemble of cities with fixed *x*. This relation does not specify the shape of *P*(*y*|*x*), e.g. it does not specify how the fluctuations $V(y|x)\equiv E(y^{2}|x)-E{\left(y|x\right)}^{2}$ of *y* around E(y∣x) scale with *x*. Here, we are interested in models *P*(*y*|*x*) satisfying

4.2 $$V(y|x)=\gamma E{\left(y|x\right)}^{\delta }.$$

This choice corresponds to Taylor’s law [24]. It is motivated by its ubiquitous appearance in complex systems [25], where typically *δ*∈[1,2], and by previous analysis of city data that reported non-trivial fluctuations [8,26,27]. The fluctuations in our models aim to effectively describe the combination of different effects, such as the variability in human activity and imprecisions on data gathering. In principle, these effects can be explicitly included in our framework by considering distinct models for each of them.

Below, we specify different models *P*(*y*|*x*) compatible with equations (4.1) and (4.2). We consider two classes of models. In the first class, which we call city models, we *a priori* choose a parametric form for *P*(*y*|*x*), and we use equations (4.1) and (4.2) to fix the free parameters. In the second class, which we call person models, we derive *P*(*y*|*x*) from a generative process for the assignment of *y* to people that is compatible with equations (4.1) and (4.2). In both cases, the likelihood L of the model is written as a function of the data {(*x*_*i*_,*y*_*i*_)}_*i*=1,…,*N*_ and at most four free parameters (*α*,*β*,*γ* and *δ*).

### 4.1. City models

In this class of models, we assume that each data point *y*_*i*_ is an independent realization from the conditional distribution *P*(*y*|*x*_*i*_) and therefore the log-likelihood can be written as

4.3 $$\ln L\equiv \ln P(y_{1},\ldots ,y_{N}|x_{1},\ldots ,x_{N})=\sum_{i=1}^{N}\ln P(y_{i}|x_{i}).$$

In order to explore how the choice of *P*(*y*|*x*) affects the outcome of the statistical analysis, we consider two different continuous distributions (Gaussian and lognormal).^1^

#### 4.1.1 Gaussian fluctuations

Here, we consider that *P*(*y*|*x*) is given by a Gaussian distribution with parameters $\mu_{N}(x)$ and $\sigma_{N}(x)$

4.4 $$P(y|x)=\frac{1}{\sqrt{2\pi }\sigma_{N}(x)}{\text{e}}^{-{\left(y-\mu_{N}(x)\right)}^{2}/2\sigma_{N}^{2}(x)}.$$

The relations (4.1) and (4.2) are fulfilled choosing the parameters as

4.5 $$\left.\begin{array}{rl} \mu_{N}(x) & =\alpha x^{\beta }\\ \;\text{and}\;\;\sigma_{N}^{2}(x) & =\gamma {\left(\alpha x^{\beta }\right)}^{\delta }. \end{array}\right\}$$

The log-likelihood (4.3) is given by

4.6 $$\ln L=\sum_{i=1}^{N}-\ln\left(\sigma_{N}(x_{i})\sqrt{2\pi }\right)-\frac{{\left(y_{i}-\mu_{N}(x_{i})\right)}^{2}}{2\sigma_{N}^{2}(x_{i})}.$$

This model has *P*(*y*≤0|*x*)>0 and therefore observations with *y*_*i*_≤0 can be accounted for. For the observables considered here, *y*=0 is a valid observation but *y*<0 is not.

We consider two cases:

*Fixed *δ*=1.* This is the typical fluctuation scaling found when *y*_*i*_ is the result of a sum of random variables [25].

*Free *δ*∈[1,2].* The general functional form that fulfils equation (4.2). We exclude *δ*>2, because, in this case, the probability *P*(*y*<0|*x*) of negative values (not feasible for most observables *y*) remains large for large *x*.

#### 4.1.2. Lognormal fluctuations

Here, we consider that *P*(*y*|*x*) is given by a lognormal distribution with parameters $\mu_{LN}(x)$ and $\sigma_{LN}(x)$:

4.7 $$P(y|x)=\frac{1}{\sqrt{2\pi }\sigma_{LN}(x)}\frac{1}{y}e^{-{\left(\ln y-\mu_{LN}(x)\right)}^{2}/2\sigma_{LN}^{2}(x)}.$$

The relations (4.1) and (4.2) are fulfilled choosing the parameters as (see appendix B)

4.8 $$\left.\begin{array}{rl} \mu_{LN}(x) & =\ln\alpha +\beta \ln x-\frac{1}{2}\sigma_{LN}^{2}(x)\\ \;\text{and}\;\;\sigma_{LN}^{2}(x) & =\ln[1+\gamma {\left(\alpha x^{\beta }\right)}^{\delta -2}]. \end{array}\right\}$$

The log-likelihood (4.3) is given by

4.9 $$\ln L=\sum_{i=1}^{N}-\ln(\sigma_{LN}(x_{i})\sqrt{2\pi })-\ln y_{i}-\frac{{\left(\ln(y_{i})-\mu_{LN}(x_{i})\right)}^{2}}{2\sigma_{LN}^{2}(x_{i})}.$$

This model has *P*(*y*≤0|*x*)=0 and therefore observations with *y*_*i*_≤0 cannot be accounted for. We again consider two cases:

*Fixed *δ*=2.* This scaling is obtained when *y*_*i*_ is the product of independent random variables. Furthermore, $\sigma_{LN}^{2}(x)$ and the fluctuations of ln⁡y are independent of *x* and therefore the maximum-likelihood estimation of *β* coincides with the estimation obtained with minimum least-squares for ln⁡y, as discussed in §3.

*Free *δ*∈[1,3].* The general functional form that fulfils equation (4.2).

### 4.2. Person model

The starting point for this class of models is the natural interpretation of equation (1.1) that people’s efficiency (or consumption) scales with the size of the city they are living in. This motivates us to consider a generative process in which tokens (e.g. a patent, a dollar of GDP, a mile of road) are produced or consumed by (assigned to) individual persons, in the same spirit as in [12,14]. Specifically, consider *j*=1,…,*M* persons living in *i*=1,…,*N* cities, on which the population of the city *i* is given by *x*_*i*_ such that $\sum_{i}^{N}x_{i}=M$. Consider also that there is a total of *k*=1,…,*Y* tokens that are randomly assigned to the *M* persons. A super-linear (sublinear) scaling suggests that a token is more likely to be assigned to someone living in a more (less) populous city. In this spirit, we assume that the probability that a token is assigned to person *j* depends only on the population *x*_(*j*)_ of the city where person *j* lives as

4.10 $$p(\;j)=\frac{x_{\left(j\right)}^{\beta -1}}{Z(\beta )},$$

where *Z*(*β*) is the normalization constant, i.e. $Z(\beta )=\sum_{j}^{M}x_{\left(\;j\right)}^{\beta -1}$. For *β*=1, *p*( *j*)=1/*M* and each person is equally likely to be assigned a token (independently of the population of its city). Equation (4.10) is a microscopic model, and we are now interested in the macroscopic behaviour of the city: the probability that a city *i* gets *y*_*i*_ tokens, given that its population is *x*_*i*_. Assuming that besides their city, individuals are indistinguishable, the probability *p*(*i*) that a token is assigned to a city *i* is given by a sum of *p*(*j*) over persons *j* on city *i*, which contains exactly *x*_*i*_ terms. Because *x*_(*j*)_=*x*_*i*_ when the person *j* lives in city *i*, represented by *j*∈*i*, we obtain

4.11 $$p(i)=\sum_{j\in i}\frac{x_{\left(j\right)}^{\beta -1}}{Z(\beta )}=\frac{x_{i}^{\beta }}{Z(\beta )}.$$

The probability of observing *y*_*i*_ tokens in each city of size *x*_*i*_ is a multinomial distribution

4.12 $$P(y_{1},\ldots ,y_{N}|x_{1},\ldots ,x_{N})=Y!\prod_{i=1}^{N}\frac{1}{y_{i}!}{\left(\frac{x_{i}^{\beta }}{Z(\beta )}\right)}^{y_{i}}.$$

Thus, the likelihood can be written as a function of the observed quantities (*x*_*i*_,*y*_*i*_) as

4.13 $$\ln L\equiv \ln P(y_{1},\ldots ,y_{N}|x_{1},\ldots ,x_{N})=\ln Y!-\sum_{i=1}^{N}\ln(y_{i}!)+\sum_{i=1}^{N}y_{i}\ln\left(\frac{x_{i}^{\beta }}{Z(\beta )}\right).$$

The scaling of the average and variance of *y*, i.e. equations (4.1), (4.2), is recovered as

4.14 $$\left.\begin{array}{rl} E(y_{i}|x_{i}) & =Yp(i)=\frac{Y}{Z_{\beta }}x_{i}^{\beta }\\ \;\text{and}\;\;V(y_{i}|x_{i}) & =Yp(i)[1-p(i)]\approx Yp(i)=E(y_{i}|x_{i}), \end{array}\right\}$$

where we identify that *α*=*Y*/*Z*(*β*), *γ*=1 and *δ*=1. For *y*_*i*_≫1, this model coincides with the city model with normal fluctuations and the latter choice of parameters. Note that the fluctuations of this model account only for fluctuations of the assignment, and neglects potential fluctuations of measurement imprecisions.

## 5. Results

In this section, we compare the models presented above against our 15 datasets. In particular, we address the following questions whose answers are summarized in table 1:

1. What is the estimated value of *β*?
  For each model, we calculate the parameters (*α*,*β*,*γ*,*δ*) that maximize L (see appendix C for details). In table 1, we report *β*.
2. What is the error bar *b* around the estimated *β*?
  We estimate *b* using bootstrapping with replacement (see appendix D for details). In table 1, *b* is shown in parentheses. The interval [*β*−*b*,*β*+*b*] can be interpreted as the 95% confidence interval of *β* when the model is not rejected. Otherwise, it can be interpreted as the robustness of the estimated *β* against fluctuations in the data (cross validation).

**Table 1.** Summary of the application of our statistical framework to 15 different databases and five models. The entries in the table represent the scaling exponent *β*. The value obtained through least-squares fitting in log-scale coincides with the value reported in the first column. The error bars were computed with bootstrap. The asterisk indicates that the model has a *p*-value higher than 0.05. If the difference *Δ*BIC between the BIC of each model with the same model with a fixed *β*=1 is below 0, the model is linear (→), between zero and six is inconclusive (open circle) and higher than six (strong evidence) is super-linear (↗)/sublinear (↘). The models were also compared between each other using the respective BICs within the same noise model (grey background has lower BIC) and between all others (bold text indicates the model with the lowest BIC).

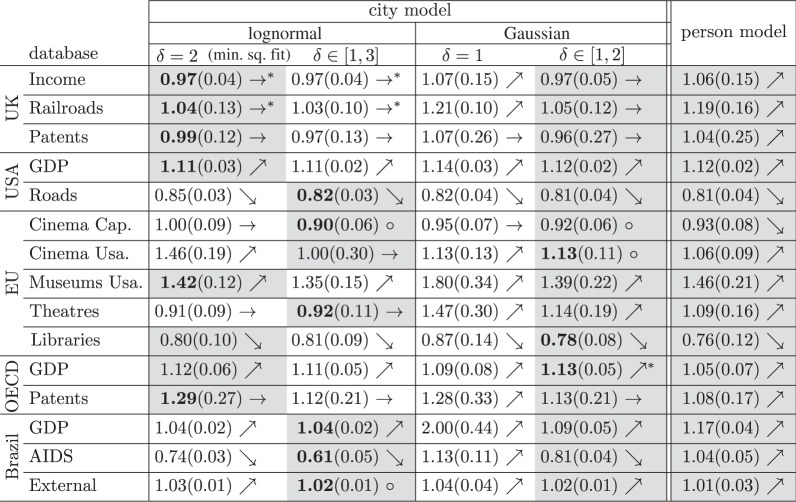

### Hypothesis testing

3. Are the data compatible with the model?
  We test the hypothesis that the data were generated by the model. Specifically, for each model, we compute a *p*-value that quantifies (i) whether the fluctuations in the data are compatible with the expected fluctuations from the model and (ii) whether the residuals are uncorrelated (see appendix E for details). In the case the model is not rejected, i.e. *p*-value >0.05, the corresponding entry in table 1 is marked with an asterisk.

### Model comparison

4. What is the statistical evidence for *β*≠1?
  We quantify the evidence for *β*≠1 by comparing the maximum-likelihood L of each model with the corresponding model where we fix *β*=1. We account for the different number of free parameters (e.g. to avoid overfitting) by using the BIC, BIC=−2lnL+kln⁡N, where *k* is the number of free parameters and *N* the number of observations (see appendix F for details). The difference in the BIC, ΔBIC≡BIC_*β*=1_−BIC_*β*_ indicates whether the model with *β*≠1 provides a sufficiently better description of the data. From this we infer that, for (i) ΔBIC<0 the model with fixed *β*=1 (linear scaling) is better, (ii) 0≤ΔBIC<6 the evidence for *β*≠1 is inconclusive, and (iii) ΔBIC≥6 the model with *β*≠1 (nonlinear scaling) is better. In table 1, these results are indicated by the symbols (i) → (linear), (ii) open circle (inconclusive), or (iii) ↘ (sublinear) or ↗(super-linear).
5. What is the statistical evidence for fluctuation scaling (Taylor’s law)?
  We quantify the evidence for *δ*≠1 (*δ*≠2), i.e. non-trivial scaling in the fluctuations in equation (4.2), in the models of cities with Gaussian (lognormal) noise. Within each class, we calculate ΔBIC≡BIC_δ*_−BIC_*δ*_, where we compare the BICs of the model where (i) *δ* is fixed (BIC_δ*_) and (ii) where *δ* is a free parameter (BIC_*δ*_). In case of ΔBIC>0, the model with *δ* as a free parameter (non-trivial fluctuation scaling) provides a better description of the data (see appendix F for details). In table 1, the entry for the selected model is highlighted with a grey background.
6. Which model best describes the data?
  We calculate the BIC of each of the five models (see appendix F for details) and select the one with the lowest BIC as the one that best describes the data. In table 1, the *β* of the selected model is printed in bold.

## 6. Discussion

In this section, we interpret the outcome of the statistical analysis summarized in table 1. We focus on specific findings and their significance to the problem of scaling in cities.

### 6.1. Data are almost never compatible with the proposed models

In almost all cases, the data are not a typical outcome of any of the five proposed models, leading to a rejection of the models (*p*-value <0.05). The only exceptions (marked by an asterisk in table 1) are the two lognormal models in UK-income and UK-train stations, and the Gaussian model with free *δ* for OECD-GDP. There are several possible reasons for the widespread rejection of the models: fluctuations of the data may differ from the fluctuations *P*(*y*|*x*) of the models (e.g. measurement errors are not correctly accounted for by *P*(*y*|*x*)); the observations are not independent (e.g. there are correlations between residuals and city size); different scalings are observed for small and large cities (as discussed in [28] and figure 3).

The rejections of the models considered here are a consequence of their strong simplifying hypothesis and show that the development of better models is needed in order to understand the observations and clarify the existence of the nonlinear scaling (1.1). It shows also that the estimated confidence interval cannot be used (in the rejected models) to discard a linear scaling *β*=1 [21]. Still, the widespread rejection of models does not imply that the nonlinear scaling (1.1) is rejected altogether because it is possible that the data are well described by another (unknown) model consistent with equation (4.1) but different from the ones considered here, e.g. having different fluctuations in *P*(*y*|*x*). These alternative models can have different fluctuation relations or can account for the known (e.g. spatial [3]) correlations in the data. In particular, the generative process underlying the person model could be generalized to account for other effects beyond city-size population (e.g. individuals could be segmented by income).

Even if most models are rejected, some models can still describe the data better than others (in terms of BIC). The conclusions drawn from such *model comparison* analysis depend on the used set of models and may change by the introduction of a better model in the future. Our investigations of scaling laws in cities in the next sections are mostly based on model comparison: we analyse which model and parameters best describe the data, with particular interest in the parameter *β*.

### 6.2. Different datasets are best described by different models

There is no single model that best describes all databases (the bold value in table 1 appears in different rows). A systematic observation on the 15 datasets is that the person model and the Gaussian model with fixed *δ* are never the best ones. This indicates that the fluctuations in the (large) cities are much larger than predicted by the scaling *δ*=1 used in both models. For the other models, there are databases in which they are the best models: the lognormal with fixed *δ*=2 is the best model in the three UK cases and for USA-GDP; the lognormal model with free *δ* is the best model for USA-roads and EU-cinema capacity; and the Gaussian model with free *δ* is the best for EU-cinema usage, OECD-GDP and EU-libraries. The inclusion of the additional parameter *δ* in the lognormal model, related to Taylor’s law in equation (4.2), is considered beneficial in eight out of the 15 cases (shaded grey regions in the two first rows of table 1). Altogether, these results show that the model underlying the usual approach (lognormal with fixed *δ*) is often not the best model.

### 6.3. The estimated *β* depends on the model

**Figure 2.**
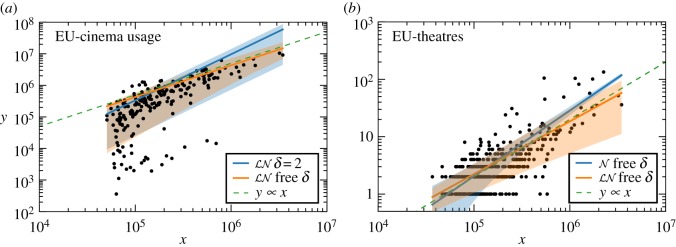
Effect of fluctuations on the estimation of *β*. (*a*) In the ‘EU cinema usage’ database, the lognormal model with *δ*=2 yields *β*=1.46, whereas free *δ* yields *β*=1.00. (*b*) In the ‘EU theatres’ database, the lognormal with free *δ* yields *β*=0.92, a lower value than *β*=1.14 obtained in the Gaussian model with free *δ*. Shaded areas represent the 68th percentile (±1 s.d.) of *P*(*y*|*x*).

Models consistent with the average scaling (4.1), but that have different assumptions regarding the fluctuations, can lead to different estimations of *β*. Consider the case of EU-cinema attendance. The value estimated from the lognormal model with fixed *δ* is *β*=1.46±0.19. It coincides with the usual approach (least-square fitting) and suggests a super-linear relation between the number of cinema visitors and the population of cities. However, if we allow for a different fluctuation scaling as in the lognormal model with free *δ*, a model that is preferred according to our BIC test, we obtain that *β*=1.00±0.30, i.e. a linear scaling. Conflicting conclusions are observed also in the EU-theatres database. The data and fittings for these two cases are shown in figure 2. Visual inspection of the graph can be misleading because of the log-scale and the different density of points, and shows the need for more careful (quantitative) statistical analysis. Altogether, the variation of *β* across different models shows that conclusions regarding *β* (e.g. *β*≠1) cannot be done independently from the analysis of the fluctuations. Considering also that different models are preferred for different databases (previous point), this confirms the practical importance of going beyond the usual approach (least-square fitting) both in terms of methods and models, as proposed in this paper.

### 6.4. Models are dominated either by the small or the large cities

The variation on the estimation of *β* across the different models can be better understood by analysing how the city size distribution shown in figure 1*a* influences the estimation of *β*. The least-square fitting minimizes the distance between the curve and the points in logarithmic scales (ln⁡y). Therefore, when data are viewed in the usual double logarithmic plot, the best curve will be the one that passes close to most points, i.e. it weights a village as much as a million-size city. The fit will be thus dominated by the large number of small cities. The disadvantage of this is that, even if the model describes well most cities, it may fail to describe the behaviour of most of the population. Our person model addresses this issue by giving the same weight to each person, leading to the problem of describing most people but potentially not most cities. To see this, consider the example of the 5565 Brazilian cities. Half of the Brazilian population lives in the 201 largest cities (3.6% of cities); yet, 50% of smallest cities account for only 8.2% of the total population. This is a direct consequence of the heavy-tailed distribution of city sizes, which holds in all our databases (figure 1*a*). Our city models with free *δ* in equation (4.2) allow cases beyond the least-squares fitting (*δ*=2) and person model (*δ*=1). The exponent *δ* controls how the variance of *P*(*y*|*x*) grows with *x*. A small variance for large *x*, obtained for small *δ*, will force the fitted curve (average) to pass close to the points of large cities. The weight of the large cities is inversely proportional to *δ*.

**Figure 3.**
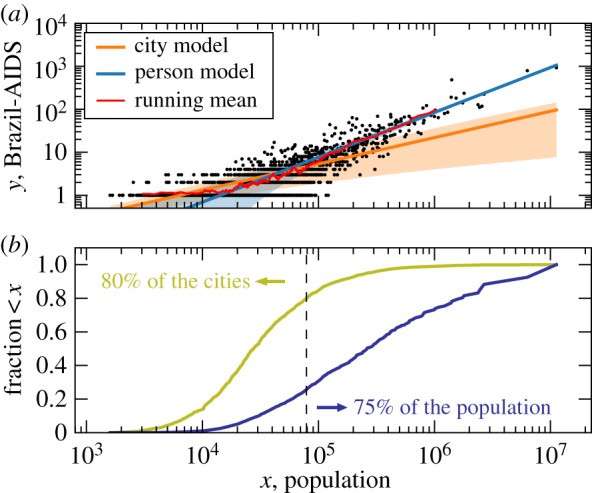
Comparison of the model of cities and persons. (*a*) Scaling of the city model, i.e. lognormal with free *δ*, and the person model (solid lines) for the data of Brazil-AIDS (dots). While the city model captures a sublinear scaling present in small cities *β*=0.61, the person model describes the roughly linear scaling *β*=1.04 of large cities. Shaded areas represent 1 s.d. The running mean (red line) is the average (〈*x*〉, 〈*y*〉) over 50 datapoints, {*x*,*y*}, in a sliding window over the data ordered in *x*. (*b*) Cumulative distribution of heavy-tailed distribution of city sizes in terms of cities and persons, i.e. the fraction of (i) cities of size ≤*x* (city model) and (ii) the population in cities of size ≤*x*.

The general considerations above explain a great extent of the variation of *β* across the models observed in table 1. The values obtained for the Gaussian model with *δ*=1 and the person model are dominated by large cities, in the lognormal *δ*=2 case, they are dominated by small cities, whereas for the free *δ* models, it depends on which best *δ* is obtained. In the Brazil-AIDS dataset *δ*≥2 and *β* is dominated by the small cities (*δ*=2 in the Gaussian model and *δ*=2.79 in the lognormal model). Accordingly, the values of *β* for these two models in the second to last row of table 1 are *β*≪1 in agreement with the lognormal with *δ*=2 case and in contrast with the Gaussian *δ*=1 and person model which have *β*>1 and are dominated by the large cities. Figure 3 shows the results for this dataset and emphasizes how different models describe different city sizes. The same reasoning also explains the values of *β* of other databases reported in table 1 (e.g. all UK cases).

In summary, the ‘weights’ each statistical model attributes to cities have an impact on the estimated value of *β* and, in particular, on the visual agreement between the data and the fit in the usual double-logarithmic plots. When the scaling relation (4.1) holds for all *x*, the difference between the models will not be significant. However, as we showed in §6.1, data are typically not compatible with models. In the cases in which *β* varies substantially across models, generalization beyond the simple scaling (1.1) [6] should be considered in order to account for the *x* dependence of *β*. In this case, the heavy-tailed distribution of city sizes leads many models to be dominated either by the large amount of small cities or by the few cities containing most of the population. This reasoning explains why cut-offs in minimum city size and aggregation of cities (different city borders) [9,16,17] influence the estimated *β*. All these procedures have a strong influence on the small cities, which are the dominant ones in the least-square fitting (e.g. aggregation of cities into metropolitan areas reduces the number of small cities). While applying cut-offs for small cities increases the visual agreement between the data and the fit in the log–log plot, this is only justified if the scaling (1.1) is interpreted as being valid only for large cities. The latter interpretation limits the relevance of the scaling because it becomes limited to a small fraction of the total cities.

### 6.5. Is the scaling nonlinear?

New answers to this central question emerge from the results of our paper (summarized in table 1). In three of the 15 cases, we found models which are reasonably compatible with the data, and we can base our conclusions on these models, i.e. on the obtained *β* and on the model comparison with the case *β*=1 (arrows →,↑,↘ in table 1). This leads to the conclusion that the UK-income and UK-train stations show linear and OECD-GDP shows super-linear scaling. In the remaining 12 cases, conclusions are based solely on model comparison, and we feel more confident to give an answer to this question only when the same conclusion is obtained for models with different fluctuations (i.e. we compare the conclusions obtained in the best model with lognormal and Gaussian fluctuations). We find such an agreement in eight of the 12 cases, so that the scalings are: UK-patents and OECD-patents are linear; USA-GDP, EU-museum usage and Brazil-GDP are super-linear; USA-roads, EU-libraries and Brazil-AIDS are sublinear. For the remaining four cases, our analysis is *inconclusive* on the question of linear or nonlinear scaling. Two reasons can lead to this conclusion. The first is that the nonlinear scaling qualitatively changes from *β*<1 to *β*>1 depending on the assumptions of the fluctuations (e.g. EU N. theatres). The second reason is that in one of the best models there is no sufficient statistical evidence for *β*≠1 (marked by an open circle in table 1, EU-cinema capacity, EU-cinema usage and Brazil-external). One interesting case falling in this second reason is EU-cinema usage, for which both the lognormal with fixed *δ* and the best model (Gaussian with free *δ*) yield *β*>1. We still consider this case inconclusive, because the best model, despite showing *β*=1.13±0.11, only marginally improves (0<BIC<6) upon the model with *β*=1. In this case, additional data are required in order to increase the statistical evidence in favour of either situation. The possibility of reaching an inconclusive answer shows the advantage of the statistical framework proposed here. In summary, in 15 datasets, we found four linear, four super-linear and three sublinear scalings.

## 7. Conclusion

In summary, we investigated the existence of non-trivial *β*≠1 scalings in city datasets. We introduced five different models, showed how to compare them and how to estimate *β*, and finally tested our methods and models in 15 different datasets. We found that in most cases models are rejected by the data and conclusions can be based only on the comparison between the descriptive power of the different models considered here. Moreover, we found that models which differ only in their assumptions on the fluctuations can lead to different estimations of the scaling exponent *β*. In extreme cases, even the conclusion on whether a city index scales linearly *β*=1 or nonlinearly *β*≠1 with city population depends on assumptions on the fluctuations. A further factor contributing to the large variability of *β* is the broad city-size distribution that makes models to be dominated either by small or by large cities. In particular, these results show that the usual approach based on least-square fitting is not sufficient to conclude on the existence of nonlinear scaling.

Recent works focused on developing generative models of urban formation that explain nonlinear scalings [10–14]. Our finding that most models are rejected by the data confirms the need for such improved models. The significance of our results on models with different fluctuations is that they show that the estimation of *β* and the development of generative models cannot be done as separate steps. Instead, it is essential to consider the predicted fluctuations not only in the validation of the model, but also in the estimation of *β*. Finally, the methods and models used in our paper can be applied to investigate scaling laws beyond cities [20,23].

## Footnotes

1 This framework also allows the use of discrete distributions.

## Data accessibility

Datasets and code used in this paper are available at http://dx.doi.org/10.5281/zenodo.49367.

## Appendix A. Databases

We used 15 datasets from five different databases. In each database (UK, USA, EU, OECD and Brazil), the same cities *x*_*i*_ were used, and the different datasets are different indexes *y*. Some of our models cannot consider *y*_*i*_≤0. In order to allow for a comparison across all models, we ignored *y*_*i*_≤0 in all cases, and below we report the number *N* of cases *y*_*i*_>0 in each dataset.

— UK: this database corresponds to fig. 5b of [17], was provided by the authors of that paper, includes the aggregation of population in cities proposed in that paper, and corresponds to the period 2000–2011.
  — Income: *N*=100, total income (weekly).
  — Train stations: *N*=97, number of train stations.
  — Patents: *N*=93, number of patents filed in the period.
— USA: this database corresponds to metropolitan areas of the USA (GDP) and urban areas (roads) in 2013. It was constructed from three different sources: the population was provided by US Census Bureau [29]; the GDP was provided by the US Bureau of Economics Analysis of the Department of Commerce [30]; and the miles of roads was provided by the US Federal Administration of Highways of the Department of Transportation (table HM-71) [31]. Similar data were used in [12].
  — GDP: *N*=381, gross domestic product of metropolitan areas.
  — Roads: *N*=459, length (in miles) of roads of urban areas.
— EU: this database is provided by Eurostat [32]. It contains population and different indexes related to culture in European cities in the year 2011.
  — Cinema capacity: *N*=418, total number of seats of cinemas.
  — Cinema usage: *N*=221, attendance of cinemas in the year.
  — Museums usage: *N*=443, attendance of museums in the year.
  — Theatres: *N*=398, number of theatres.
  — Libraries: *N*=597, number of public libraries.
— OECD: this database contains indexes of cities from the Organization for Economic Co-operation and Development in the years 2000–2012 [33].
  — GDP: *N*=275, gross domestic product in 2010.
  — Patents: *N*=218, number of patents filed in 2008.
— Brazil: this database contains different indexes of all municipalities of Brazil. The data are from the year 2010 and are provided by Brazil’s Health Ministry (Brazilian Health Ministry. July 2015; population corresponds to census data).
  — GDP: *N*=5565, gross domestic product.
  — AIDS: *N*=1812, number of deaths by AIDS.
  — External: *N*=5286, number of deaths by external causes.

All the above databases are provided at http://dx.doi.org/10.5281/zenodo.49367.

## Appendix B. Taylor’s law in lognormal

Here, we express the parameters of the lognormal distribution, $\mu_{LN}(x)$ and $\sigma_{LN}^{2}(x)$, as a function of the parameters of the scaling laws

4.1 $$E(y|x)=\alpha x^{\beta }$$

and

4.2 $$V(y|x)=\gamma E{\left(x\right)}^{\delta },$$

*α*,*β*,*γ* and *δ*. Noting that the expectation and the variance of the lognormal distribution, equation (4.7), are given by

B 1 $$E(y|x)={\text{e}}^{\mu_{LN}(x)+\sigma_{LN}^{2}(x)/2}$$

and

B 2 $$V(y|x)=({\text{e}}^{\sigma_{LN}^{2}(x)}-1)E{\left(y|x\right)}^{2},$$

we find a unique solution for $\mu_{LN}(x)$ and $\sigma_{LN}^{2}(x)$ by comparing with equations (4.1) and (4.2):

4.8 $$\left.\begin{array}{rl} \mu_{LN}(x) & =\ln\alpha +\beta \ln x-\frac{1}{2}\sigma_{LN}^{2}(x)\\ \;\text{and}\;\;\sigma_{LN}^{2}(x) & =\ln[1+\gamma {\left(\alpha x^{\beta }\right)}^{\delta -2}]. \end{array}\right\}$$

## Appendix C. Maximization of the likelihood

The maximization of the likelihood is performed by minimizing minus the log-likelihood, using the algorithm ‘L-BFGS-B’ [34], whose implementation can be found on the Python package SciPy [35], and the details can be found at http://dx.doi.org/10.5281/zenodo.49367. Given that the minimization algorithm can converge to a local minimum, our procedure repeats the optimization 512 times, each with random initial parameters, and selects the lowest. We confirmed that increasing from 256 to 512 samples did not change the computed minima, an indication that the algorithm found the global one.

## Appendix D. Computation of the error estimates

The error estimates were computed using bootstrap [36]. The method consists of sampling *N* pairs (*x*_*i*_,*y*_*i*_) with replacement from the set of *N* available data points, and repeating the maximization procedure outlined in the previous section for each set. This procedure (*sampling*+*maximization*) was repeated 100 times for each combination (model, dataset) and the error estimates were computed as the standard deviation of the distances from the measured parameters to the estimated parameter from the true dataset. We confirmed that the bootstrap error estimates for the lognormal fixed-*δ* case are within 1% equal to the values of the least-square fit.

## Appendix E. Computation of the *p*-value

The computation of the *p*-value was done by defining a statistic that tests the hypothesis used in each model; in the case of the lognormal and normal models these are (i) data are independent and (ii) the data are compatible with the model. We used a statistic based on the D’Agostino *K*^2^ test [37] (over ln⁡y or *y*, respectively) that computes the deviations from 0 of the empirical kurtosis and skewness; the test consist of comparing it with the fluctuations expected from a finite-size sample from the (null) model. In detail, we compute two statistics, *Z*_*s*_ and *Z*_*k*_ for the kurtosis and skewness, respectively. Each of them has a $\chi_{1}^{2}$ distribution under the null hypothesis, so the sum $K^{2}=Z_{s}^{2}+Z_{k}^{2}$ has a $\chi_{2}^{2}$ distribution (with 2 degrees of freedom). Because this test does not test independence of the samples, we include in the test statistic the Spearman’s rank correlation [38] of the residuals of the fit, *Z*_*S*_ (also distributed as a $\chi_{1}^{2}$) because if the residuals are correlated, the data are not independent. The *p*-value is thus computed by measuring how extreme $K^{2}=Z_{s}^{2}+Z_{k}^{2}+Z_{S}^{2}$ is in the $\chi_{3}^{2}$ distribution (with 3 degrees of freedom). The implementation of this is available at http://dx.doi.org/10.5281/zenodo.49367.

In the population model, the calculation of the *p*-value must be different, because the variance is not being left as a free parameter, so we take a more *classical* approach. The *p*-value is computed by measuring how extreme is the difference between the data and its fit with respect to the difference between a sample from the model and its fit. In practice, we use a *χ*^2^ statistic to measure the distance between two sets of points {*y*_*i*_}_*i*_ (data) and {*m*_*i*_}_*i*_ (the model), $\chi^{2}=\sum_{i}{\left(y_{i}-m_{i}\right)}^{2}/y_{i}$. Then, we generate from the model 200 different samples. For each of these samples, we compute the *χ*^2^ between the sample values and their fits. Finally, we compute the *p*-value as the fraction of samples whose *χ*^2^ is bigger than the one that belongs to the real data. Note that this statistic is not taking into account independence of the residuals (if we consider the multinomial distribution as the null model, then they should not be independent) or normality in the strict sense, so this test is more permissive than the previous.

## Appendix F. Model comparison using Bayesian information criterion

We compare two models *m*=1,2 by calculating the BIC [39], ${\mathrm{BIC}}_{m}\equiv -2\ln L_{m}+k_{m}\ln N$, where *N* is the number of data points (observations), $L_{m}$ is the maximum-likelihood of the model, and *k*_*m*_ is the number of estimated (free) parameters of the model. In this approach, the model with a lower value for the BIC gives a better description of the data.

We can quantify how much better one model compares with the other by looking at the Bayes factor [40], *B*_12_=*P*(data|*m*=1)/*P*(data|*m*=2), where *P*(data|*m*) is the evidence for model *m*, i.e. the probability of the data given the model. It can be shown [36] that this quantity can be approximated by

F 1 $$B_{12}\approx {\text{e}}^{\Delta \mathrm{BIC}/2},$$

where ΔBIC≡BIC_2_−BIC_1_ is the difference of the respective BICs. Thus, if BIC_1_<BIC_2_, it follows that *B*_12_>1, i.e. that model 1 provides a better description of the data than model 2. Regarding the decision about nonlinear scaling, i.e. *β*≠1, we require that ΔBIC≡BIC_*β*=1_−BIC_*β*_≥6 (see main text), in line with [40], where it is suggested that this implies strong or very strong evidence for a model with *β*≠1. This corresponds to *B*_12_≥*e*^3^≈20.08, i.e. it is at least 20 times more likely that the data come from a model with *β*≠1.

## References

[1] Batty M 2013 *The new science of cities*. Cambridge, MA: MIT Press.

[2] Bettencourt LMA, Lobo J, Helbing D, Kuhnert C, West GB 2007 Growth, innovation, scaling, and the pace of life in cities. *Proc. Natl Acad. Sci. USA* 104, 7301–7306. (doi:10.1073/pnas.0610172104) 17438298 10.1073/pnas.0610172104 PMC1852329

[3] Bettencourt LMA, Lobo J, Strumsky D, West GB 2010 Urban scaling and its deviations: revealing the structure of wealth, innovation and crime across cities. *PLoS ONE* 5, e13541 (doi:10.1371/journal.pone.0013541) 21085659 10.1371/journal.pone.0013541 PMC2978092

[4] Arbesman S, Christakis NA 2011 Scaling of prosocial behavior in cities. *Physica A, Stat. Mech. Appl.* 390, 2155–2159. (doi:10.1016/j.physa.2011.02.013) 10.1016/j.physa.2011.02.013 PMC3359052 22639486

[5] Gomez-Lievano A, Youn H, Bettencourt LMA 2012 The statistics of urban scaling and their connection to Zipf’s law. *PLoS ONE* 7, e40393 (doi:10.1371/journal.pone.0040393) 22815745 10.1371/journal.pone.0040393 PMC3399879

[6] Bettencourt LMA, Lobo J, Youn H 2013 The hypothesis of urban scaling: formalization, implications and challenges. (http://arxiv.org/abs/1301.5919)

[7] Alves LGA, Ribeiro HV, Lenzi EK, Mendes RS 2013 Distance to the scaling law: a useful approach for unveiling relationships between crime and urban metrics. *PLoS ONE* 8, e69580 (doi:10.1371/journal.pone.0069580) 23940525 10.1371/journal.pone.0069580 PMC3734155

[8] Nomaler Ö, Frenken K, Heimeriks G 2014 On scaling of scientific knowledge production in U.S. metropolitan areas. *PLoS ONE* 9, e110805 (doi:10.1371/journal.pone.0110805) 25353686 10.1371/journal.pone.0110805 PMC4212974

[9] Oliveira EA, Andrade JS Jr, Makse HA 2014 Large cities are less green. *Sci. Rep.* 4, 4235 (doi:10.1038/srep04235) 24577263 10.1038/srep04235 PMC3937786

[10] Samaniego H, Moses ME 2008 Cities as organisms: allometric scaling of urban road networks. *J. Transport Land Use* 1, 21–39. (doi:10.5198/jtlu.v1i1.29)

[11] Um J, Son S-W, Lee S-I, Jeong H, Kim BJ 2009 Scaling laws between population and facility densities. *Proc. Natl Acad. Sci. USA* 106, 14 236–14 240. (doi:10.1073/pnas.0901898106) 10.1073/pnas.0901898106 PMC2732869 19706506

[12] Bettencourt LMA 2013 The origins of scaling in cities. *Science* 340, 1438–1441. (doi:10.1126/science.1235823) 23788793 10.1126/science.1235823

[13] Pan W, Ghoshal G, Krumme C, Cebrian M, Pentland A 2013 Urban characteristics attributable to density-driven tie formation. *Nat. Commun.* 4, 1961 (doi:10.1038/ncomms2961) 23736887 10.1038/ncomms2961

[14] Yakubo K, Saijo Y, Korošak D 2014 Superlinear and sublinear urban scaling in geographical networks modeling cities. *Phys. Rev. E* 90, 022803 (doi:10.1103/PhysRevE.90.022803) 10.1103/PhysRevE.90.022803 25215777

[15] Shalizi CR 2011 Scaling and hierarchy in urban economies. (http://arxiv.org/abs/1102.4101)

[16] Louf R, Barthelemy M 2014 Scaling: lost in the smog. *Environ. Plan. B: Plan. Des.* 41, 767–769. (doi:10.1068/b4105c)

[17] Arcaute E, Hatna E, Ferguson P, Youn H, Johansson A, Batty M 2015 Constructing cities, deconstructing scaling laws. *J. R. Soc. Interface* 12, 20140745 (doi:10.1098/rsif.2014.0745) 25411405 10.1098/rsif.2014.0745 PMC4277074

[18] Bettencourt LMA, Lobo J 2015 Urban scaling in Europe. (http://arxiv.org/abs/1510.00902)

[19] Rybski D 2013 Auerbach’s legacy. *Environ. Plan. A* 45, 1266–1268. (doi:10.1068/a4678)

[20] Savage VM, Gillooly JF, Woodruff WH, West GB, Allen AP, Enquist BJ, Brown JH 2004 The predominance of quarter-power scaling in biology. *Funct. Ecol.* 18, 257–282. (doi:10.1111/j.0269-8463.2004.00856.x)

[21] Thulin M 2014 On confidence intervals and two-sided hypothesis testing. PhD thesis, Uppsala University, Sweden.

[22] Zar JH 1968 Calculation and miscalculation of the allometric equation as a model in biological data. *Bioscience* 18, 1118–1120. (doi:10.2307/1294589)

[23] Warton DI, Wright IJ, Falster DS, Westoby M 2006 Bivariate line-fitting methods for allometry. *Biol. Rev.* 81, 259–291. (doi:10.1017/S1464793106007007) 16573844 10.1017/S1464793106007007

[24] Taylor LR 1961 Aggregation, variance and the mean. *Nature* 189, 732–735. (doi:10.1038/189732a0)

[25] Eisler Z, Bartos I, Kertész J 2008 Fluctuation scaling in complex systems: Taylor’s law and beyond. *Adv. Phys.* 57, 89–142. (doi:10.1080/00018730801893043)

[26] Hanley QS, Khatun S, Yosef A, Dyer R-M 2014 Fluctuation scaling, Taylor’s law, and crime. *PLoS ONE* 9, e109004 (doi:10.1371/journal.pone.0109004) 25271781 10.1371/journal.pone.0109004 PMC4182799

[27] Greig A, Dewhurst J, Horner M 2015 An application of Taylor’s power law to measure overdispersion of the unemployed in English labor markets. *Geogr. Anal.* 47, 121–133. (doi:10.1111/gean.12046)

[28] Hanley QS, Lewis D, Ribeiro HV 2016 Rural to urban population density scaling of crime and property transactions in English and Welsh parliamentary constituencies. *PLoS ONE* 11, e0149546 (doi:10.1371/journal.pone.0149546) 26886219 10.1371/journal.pone.0149546 PMC4757021

[29] US Census Bureau. 2014 See www.census.gov/popest/data/metro/totals/2014/.

[30] US Bureau of Economics Analysis. 2015 See www.bea.gov/itable/index_regional.cfm.

[31] US Department of Transportation. 2015 See www.fhwa.dot.gov/policyinformation/statistics/2013/.

[32] Eurostat. 2015 See http://ec.europa.eu/eurostat/web/cities/data/database.

[33] OECD. 2015 See http://dx.doi.org/10.1787/data-00531-en.

[34] Byrd RH, Lu P, Nocedal J, Zhu C 1995 A limited memory algorithm for bound constrained optimization. *SIAM J. Sci. Comput.* 16, 1190–1208. (doi:10.1137/0916069)

[35] Jones E, Oliphant T, Peterson P 2001 SciPy: open source scientific tools for Python. See http://www.scipy.org.

[36] Hastie T, Tibshirani R, Friedman J 2009 *The elements of statistical learning*, 2nd edn Springer Series in Statistics New York, NY: Springer.

[37] D’Agostino RB 1986 *Goodness-of-fit-techniques*. New York, NY: Marcel Dekker.

[38] Kendall MG 1970 *Rank correlation methods*, 4th edn. London, UK: Griffin.

[39] Schwarz G 1978 Estimating the dimension of a model. *Ann. Stat.* 6, 461–464. (doi:10.1214/aos/1176344136)

[40] Kass RE, Raftery AE 1995 Bayes factors. *J. Am. Stat. Assoc.* 90, 773–795. (doi:10.1080/01621459.1995.10476572)

